# How to allocate intensive care resources during the COVID-19 pandemic: medical triage or *a priori* selection?

**DOI:** 10.3325/cmj.2020.61.276

**Published:** 2020-06

**Authors:** Ante Sekulić, Robert Likić, Marijana Matas

**Affiliations:** 1 University of Zagreb School of Medicine, Zagreb, Croatia; 2 Department of Anesthesiology and Intensive Care, Clinical Hospital Centre Zagreb, Zagreb, Croatia; 3 Division of Clinical Pharmacology and Therapeutics, Clinical Hospital Centre Zagreb, Zagreb, Croatia *rlikic@kbc-zagreb.hr*

The pandemic outbreak of coronavirus disease 2019 (COVID-19) has seriously affected social life, economy, and medical community around the world. One of the complications of this viral infection is acute respiratory failure, a serious condition treated by artificial blood oxygenation and carbon dioxide removal. These treatments require the use of modalities based on complex and expensive health technology, such as mechanical ventilation of the lungs, respiratory dialysis, extracorporeal membrane oxygenation, hyperbaric oxygenation, and blood filtering. A sharp increase in the number of infected patients worldwide has created acute shortages of medical technology for intensive care life support (1). Consequently, not all patients who need a full intensive care treatment have access to it. In such circumstances, fair allocation of resources remains a very challenging task, which has been approached from various ethical perspectives (2,3). Here, we discuss the ethical principles used in this setting based on the literature data and experiences gained during the first two months of the COVID-19 pandemic in Croatia.

Ethical consideration of resource allocation in intensive care medicine takes into account several important factors: cost-effectiveness analyses, severity of illness assessment, and prediction of treatment outcome.

## Cost-effectiveness analyses

Resource allocation in intensive care medicine is a decision-making process, unavoidable part of which are cost-effectiveness analyses. Hospital treatment is expensive, and the costs of intensive care amount to around 20% of the total hospital expenditure (4,5). Moreover, during the ICU stay, mechanical ventilation of the lungs could increase the daily costs by additional 26% (6). In the USA, ventilatory support for a COVID-19 patient lasting longer than 96 hours has an estimated median cost of $ 88 114. Other factors associated with intensive care still await to be evaluated, especially in the setting of a widespread infection outbreak (7). It has been reported that ICUs reduce the mortality rate in a cost-effective manner, and cost-effectiveness analyses are a strong argument in resource allocation for intensive care medicine (8). Nevertheless, a relatively new publication concluded that “despite critical care being a significant healthcare cost burden, there is a paucity of studies evaluating its cost effectiveness” (9).

## Severity of illness and admission to the intensive care unit

Resource allocation to patients who are severely ill and have the best possibility for a good recovery is discussed in many articles. Instruments for severity of illness assessment are based on statistical methods and large data sets analyses, while knowledge and experience of the attending physicians are presumed. Numerous scoring systems have been developed for admission to the intensive care unit, each with different performance characteristics. Additionally, special scoring systems have been devised to guide the decision-making process related to the admission of patients with community acquired pneumonias to intensive care units (10). This is a topic of ongoing research, and additional studies are required.

## Outcome prediction

Outcome prediction in COVID-19 patients is based on newly developed scoring systems (11). Even though numerous models were analyzed, the majority were poorly reported, with a high risk of bias. Only one study involved patients outside of China. For now, all reported prediction models can at best be considered as a basis for further modeling.

## Consideration of ethical dilemmas

Discussing ethical dilemmas based on the aforementioned arguments entails a great deal of uncertainty. The conclusions about cost-effectiveness, severity of illness as a factor of assessment for intensive care admission, and predictions of treatment outcome all remain methodologically insufficient. Severity of illness and outcome prediction are based on biological parameters, which cannot be used in resolving ethical dilemmas.

Ethical dilemmas usually arise as a result of economic issues, eg, a shortage of fully operated mechanical ventilators and available intensive care beds. Who will be admitted to an intensive care unit? Who will get a ventilator? When to stop the treatment? Can the ventilator be taken from one patient and given to another one with better chances of survival and good outcome? Some of these dilemmas were recognized almost half a century ago, but the debate is still ongoing (12). The complexity of ethical dilemmas during the COVID-19 outbreak has been recently recognized worldwide, particularly in Italy (13).

Decision-making process in medicine can lead to frustrating and dramatic situations. Textbooks on intensive care describe several strategies of resource allocation: autocracy, democracy, equality, lottery, capitalism, personal worth, and utilitarianism (14). During the COVID-19 pandemic, two opposing ethical approaches have emerged: medical triage and *a priori* selection of patients (15). Medical triage is based on the severity of illness, while *a priori* selection of patients is based on the predictions of the possibility for a good outcome of the ICU treatment. Medical triage focuses on one patient (the most severely ill one) and *a priori* selection focuses on a group of patients meeting certain criteria. In this way, the ethical principle of justice remains confronted with the utilitarian ethics. While medical triage attempts to save a life, *a priori* selection tries to save life-years. Younger patients can achieve more savings in life-years. Thus, the patient selection goes against the ethical principle of equity. *A priori* selection of patients, based on utilitarian ethics, stresses the quality of life after intensive care treatment as an important selection criterion, which is a questionable argument since quality of life after intensive care could be poor and mortality rate high (16).

Medical triage works until an explosion in new cases overruns all ICU capacities. After that point, the ethical approach has to be replaced by utilitarian ethical attitude. The selection of patients with better chances of a good outcome entails some serious disadvantages: not only is the ethical principle of equity absent, but the principle of the patient’s autonomy is also missing. An exception remains a rare anecdotal report about an older Italian priest, don Giuseppe Berardelli, who gave his ventilator to a younger patient (17).

Furthermore, a shortage of medical equipment can create another disastrous situation: transfer of a single ventilator from an older patient to a younger one. Taking away technology from one patient in order to save another one (who presumably has better chances of a good outcome) means taking a life away. No one has the right to do this since outcome prediction in intensive care medicine is still not an exact science. While medical triage requires skilled intensivists, numerous published guidelines cannot replace distributive justice (18).

So, where is the difference between these two ethical approaches? Medical triage respects the essential human right to life no matter how old or severely ill someone is. In contrast, patient selection is based on age, comorbidities, and predictions of treatment outcome. These arguments are not established on hard science and violate the ethical principle of fair resource allocation during intensive care treatment.

International law recognizes 30 essential human rights (19). The most prominent among them is the right to live and to be healthy. There is also the right to receive the best medical help available. These points of international law are usually omitted in discussions about ethical dilemmas and resource allocation in intensive care medicine.

## Experiences from Croatia

Intensive care resources in Croatia were significantly upgraded by building military camps and additional intensive care capacity for the treatment of COVID-19 patients. The initial shortage of protective equipment was eased by imports from abroad, while successful epidemiological measures prevented an exponential increase in new COVID-19 cases. Under such circumstances, medical triage of patients has remained a valid ethical approach.

## Conclusion

During the current pandemic, achieving balance between the number of newly detected and severely ill patients and the number of available intensive care beds mandates the application of an ethical approach. Medical triage of patients is a valid ethical strategy that is fully in accord with international law when there is no exponential growth of newly detected cases. National health authorities should do their utmost to manage and control the epidemic dynamics in order to prevent the exponential growth of newly diagnosed and severely ill cases. In this regard, Croatia has so far been successful.
